# Granulin Exacerbates Lupus Nephritis via Enhancing Macrophage M2b Polarization

**DOI:** 10.1371/journal.pone.0065542

**Published:** 2013-06-05

**Authors:** Xi Chen, Zhenke Wen, Wei Xu, Sidong Xiong

**Affiliations:** 1 Institute for Immunobiology, Shanghai Medical College, Fudan University, Shanghai, People’s Republic of China; 2 Institutes of Biology and Medical Sciences, Soochow University, Suzhou, People’s Republic of China; University Paris Sud, France

## Abstract

**Background and Aims:**

Lupus nephritis (LN), with considerable morbidity and mortality, is one of the most severe manifestations of systemic lupus erythematosus (SLE). Yet, the pathogenic mechanisms of LN have not been clearly elucidated, and efficient therapies are still in great need. Granulin (GRN), a multifunctional protein linked to inflammatory diseases, has recently been reported to correlate with the disease activity of autoimmune diseases. However, the role of GRN in the pathogenic process of LN still remains obscure. In this study, we explored its potential role and underlying mechanism in the pathogenesis of LN.

**Methodology/Principal Findings:**

We found that serum GRN levels were significantly up-regulated and were positively correlated with the severity of LN. Overexpression of GRN *in vivo* by transgenic injection remarkably exacerbated LN, whereas down-regulation of GRN with shRNA ameliorated LN, firmly demonstrating the critical role of GRN in the pathogenesis of LN. Notably, macrophage phenotype analysis revealed that overexpression of GRN could enhance macrophage polarization to M2b, a key mediator of the initiation and progression of LN. On the contrary, down-regulation of GRN resulted in impaired M2b differentiation, thus ameliorating LN. Moreover, we found that MAPK signals were necessary for the effect of GRN on macrophage M2b polarization.

**Conclusion/Significance:**

We first demonstrated that GRN could aggravate lupus nephritis (LN) via promoting macrophage M2b polarization, which might provide insights into the pathogenesis of LN as well as potential therapeutic strategies against LN.

## Introduction

Systemic lupus erythematosus (SLE), a chronic inflammatory autoimmune disorder, is a potentially fatal disease characterized by immune complex deposition and the subsequent inflammation that contributes to sever tissue damage [1]. One of the most severe manifestations is lupus nephritis (LN), which remains a cause of substantial morbidity and mortality. LN occurs in up to 50% of patients at onset of the disease and over 60% of patients during the progression of SLE [2]. Recently reported 10-year survival rates of patients with lupus nephritis range from 68% to 98.2% [3]. A better understanding of the pathogenesis of LN is an important step in identifying more targeted therapeutic approaches. Substantial researches have helped define the pathogenic mechanisms of renal manifestations. Immune complex (IC) glomerular deposits generate release of proinflammatory cytokines and chemokines causing inflammation, leading to monocytes and polymorphonuclear cells chemotaxis. Subsequent release of proteases generates endothelial injury and mesangial proliferation. And then, the presence of ICs also promotes adaptive immune response and causes release of type I interferon which further activates macrophages to release more proinflammatory molecules, resulting in epithelial glomerular proliferation and fibrosis [4]. However, underlying molecular mechanisms that mediate LN still remain unclear so far, thus impeding the advance of efficient therapies toward LN.

Recent reports showed that some multifunctional proteins, which were previously studied for their roles in autoimmune and inflammatory diseases, might be involved in the pathogenesis of LN [5]. A promising candidate is granulin (GRN), a glycosylated protein with a repeating cysteine-rich motif [6], is highly expressed in epithelial cells, certain types of neurons, and macrophages [7]. GRN is originally identified as an autocrine growth factor that regulates cell growth, development, and tissue remodeling [8]–[10]. As a multifunctional protein, GRN has also been linked to a variety of physiologic and disease processes, including inflammation, wound healing and regulation of innate immunity [11]–[13]. Furthermore, recent studies have shown that GRN is correlated with autoimmune diseases, including rheumatoid arthritis, multiple sclerosis and type-2 diabetes [14]–[18]. And one report has found that GRN is associated with the disease activity of SLE [19]. However, whether GRN takes responsibility in the pathogenic mechanisms of LN still remains unclear.

Our previous study has demonstrated that syngeneic activated lymphocyte derived DNA (ALD-DNA) could function as an auto-antigen to induce SLE syndrome including severe renal manifestations in syngeneic BALB/c mice [20]–[26]. Given the emblematical autoimmune syndrome and exclusion of the genetic mutation interruptions in this lupus model, the ALD-DNA-induced lupus mice could be used as an ideal model to explore the pathogenic mechanisms for LN.

Here, we carefully determined the potential role and possible mechanism of GRN in the pathogenesis of LN using ALD-DNA-induced lupus model. We demonstrated that serum GRN levels were correlated with the severity of LN. Moreover, up-regulation of GRN could exacerbate LN, whereas down-regulation of GRN could ameliorate LN. Interestingly, we found that GRN aggravated LN via facilitating ALD-DNA-induced macrophage M2b polarization. And we also found that MAPK signaling pathways were required for GRN to potentiate ALD-DNA-induced macrophage M2b polarization. Collectively, these results indicated that GRN exacerbated LN via promoting M2b polarization, which might provide a novel mechanism accounting for the progression of LN and a clue for developing novel therapeutic strategies against LN.

## Materials and Methods

### Ethics Statement

This study was strictly carried out according to the Guide for the Care and Use of Medical Laboratory Animals (Ministry of Health, P.R. China, 1998) and with the ethical approval of the Shanghai Medical Laboratory Animal Care and Use Committee (Permit number: SYXK 2010-0036) as well as the Ethical Committee of Fudan University (Permit number: 2010016). All surgery was performed under sodium pentobarbital anesthesia, and all animal procedures in this study were strictly performed in a manner to minimize suffering of laboratory mice.

### Mice and Cell Culture

Six-week-old female BALB/c mice were purchased from the Experimental Animal Center of Chinese Academy of Sciences (Shanghai, P. R. China). Mice were housed in a specific pathogen free room under controlled temperature and humidity. Primary peritoneal macrophages were obtained from six-week-old female BALB/c mice and were maintained in DMEM (Invitrogen Life Technologies) supplemented with 10% FBS (Invitrogen Life Technologies) in a 5% CO_2_ incubator at 37°C.

### Reagents and Antibodies

Elastase inhibitor was purchased from Sigma. Recombinant mouse granulin was from Sino Biological Inc. (Beijing, China). MEK1/2 inhibitor U0126, as well as p38 MAPK inhibitor SB203580, was purchased from Calbiochem. JNK inhibitor SP600125 was from Santa Cruz Biotechnology. The granulin specific siRNA (acrogranin siRNA) and Control siRNA-A were purchased from Santa Cruz Biotechnology. Macrophages were transfected with 200 nM of the above RNAs as indicated, by Mouse Macrophage Nucleofector Kit (Lonza) according to the manufacturer’s instruction. Phospho-Akt, ERK1/2, SAPK/JNK, p38 antibodies and iNOS antibody were from Cell Signaling Technology, and anti-β-actin antibody was from Santa Cruz Biotechnology. Granulin antibody was obtained from R&D Systems.

### Plasmids Construction and Lentivirus Production

The full length of GRN cDNA was amplified from total RNA of RAW264.7 cells using the primers 5′-CGGCTAGCGCTAATGGAAATTGAGGTGGGC-3′ and 5′-CCCAAGCTTCTT- ACAGTAGCGGTCTTGGCCAAGACCGCTACTGTAAG-3′. After digested by Nhe I and Hind III, the resulting PCR product was then subcloned into pcDNA3.1. The GRN shRNA oligos were inserted into pLKO.1 (Addgene) to generate pLKO.1-shGRN that contained the shRNA targeting mouse GRN (5′-ACTCATCCTGAGTCACCCTAT-3′). The recombinant pseudo-typed lentivirus was generated by co-transfection of three plasmids pLKO.1-shGRN/pLKO.1-nontargeting shRNA, psPAX2 and pMD2.G (Addgene) into 293T cells using Lipofectamine Plus (Invitrogen), concentrated by ultracentrifugation and stored at −80°C as previously described [27].

### Measurement of Lentiviral Titers

We measured lentivirus titers by the assessment of vector DNA in transduced cells (DNA titers), which was more rigorous and reliable than RNA titers and GFP titers [28]. Forward primer for lentivirus vector (5′-ACCTGAAAGCGAAAGGGAAAC-3′) and reverse primer for lentivirus vector (5′-CACCCATCTCTCTCCTTCTAGCC-3′) were used for real-time PCR. For determining DNA titers, genomic DNA from approximately 1–10×10^6^ vector transduced 293T cells was isolated 4 days after transduction using the Puregene kit (Promega, Madison, WI, USA). DNA was isolated from cells transduced with several dilutions of vector. Genomic DNA was subjected to quantitative real-time PCR using a Lightcycler480 and SYBR Green system (Roche Diagnostic Systems) following the manufacturer’s protocol. All reactions were carried out in triplicate. Amplification of vector plasmid DNA for generation of standard curves was performed using concentrations of plasmids ranging from 10^7^ molecules/µL to 10 molecules/µL as determined by spectrophotometry. Plasmid DNA used for standard curve generation was diluted with genomic DNA from uninfected 293T cells to control for any inhibitory effect of genomic DNA on PCR. The number of vector DNA molecules in transduced cells was calculated by comparing threshold cycle (Ct) values of samples to that of the plasmid standard curve. For determining the final DNA titer of vectors, the total number of vector DNA molecules in transduced cells was corrected for dilution and the number of cells plated and infected [28].

### DNA Preparation

ALD-DNA and UnALD-DNA were prepared with murine splenocytes which were generated from surgical resected spleens of six- to eight-week-old female BALB/c mice and cultured with or without Con A (Sigma-Aldrich) *in vitro* as previously described [21]. Briefly, for generation of ALD-DNA, splenocytes were seeded at 2×10^6^ cells/mL in 75 cm^2^ cell culture flask and cultured in the presence of Con A (5 µg/mL) for 6 days to induce apoptosis. The apoptotic cells were stained with FITC-labeled Annexin V (BD Biosciences) and propidiumiodide (PI; Sigma-Aldrich), and sorted using a FACSAria (BD Biosciences). Genomic DNAs from syngeneic apoptotic splenocytes were treated with S1 nuclease (TaKaRa) and proteinase K (Sigma-Aldrich), and then purified using the DNeasy Blood & Tissue Kits (Qiagen) according to the manufacturer’s instructions. UnALD-DNA was prepared with unactivated (resting) splenocytes and extracted using the same methods. To exclude contamination with LPS, sterile endotoxin-free plastic ware and reagents were used for DNA preparation. DNA samples were also monitored for low level of endotoxin by the Limulus amoebocyte lysate assay (BioWhittaker) according to the manufacturer’s instructions. The concentration of DNA was determined by detection of the absorbance (A) at 260 nm. The apoptotic DNA ladder of ALD-DNA was confirmed by agarose gel electrophoresis (AGE) [20]–[22], [24]–[26].

### Generation of Lupus Model

To generate lupus model, 6- to 8-wk-old syngeneic female BALB/c mice were divided into several groups of 8 mice and actively immunized by subcutaneous injection on the back with 0.2 mL of an emulsion containing ALD-DNA (50 µg/mouse) in phosphate-buffered saline (PBS) plus equal volume of complete Freund’s adjuvant (CFA; Sigma-Aldrich) at week 0, and followed by two booster immunizations of ALD-DNA (50 µg/mouse) emulsified with IFA (Sigma-Aldrich) at week 2 and week 4 for total 3 times as previously described [20], [21]. 8 mice in each group received an equal volume of PBS plus CFA or IFA, or UnALD-DNA (50 µg/mouse) plus CFA or IFA were used as controls. Mice were bled from retro-orbital sinus prior to injection and at 2-week intervals until 3 months after the initial injection. Urine was also collected at 2-week intervals until 3 months after the initial injection. 8 or 12 weeks later, mice were humanely sacrificed and surgical resected kidneys were collected for further analysis.

### pGRN Treatment and Lentivirial Delivery in Mice

To examine the potential role of GRN, 8 mice in each group were intramuscularly injected with 100 µg per mouse pcDNA3.1-GRN (pGRN) or pcDNA3.1 as control 72 h earlier before injection with ALD-DNA. And mice were then injected with pGRN every 2 weeks for total 6 times [24]. Approximately 2×10^8^ molecules of lentivirus encoding shRNA directed against GRN (LV-shGRN) per mouse were delivered into 8 mice through intravenous injection 72 h before the establishment of lupus model and lentivirus encoding nontargeting shRNA (LV-shNC) was used as control as previously reported [27].

### Autoantibody and Proteinuria Examination

Anti-dsDNA antibodies in the mice serum were determined by ELISA assay as described previously [20]. In brief, ELISA plates (Costar) were pretreated with protamine sulphate (Sigma-Aldrich) and then coated with calf thymus dsDNA (Sigma-Aldrich). After incubation with mouse serum, the levels of anti-dsDNA Abs were detected with the horseradish peroxidase (HRP)-conjugated goat anti-mouse IgG (Southern Biotech). Tetramethylbenzidine (TMB) substrate was used to develop colors and absorbance at 450 nm was measured on a microplate reader (BIO-TEK ELX800). Proteinuria of the urine samples was measured with the BCA Protein Assay Kit (Thermo Fisher Scientific) according to the manufacturer’s instructions as previously described [22], [24], [25]. Prepare working reagent (WR) by mixing 50 parts of BCA Reagent A with 1 part of BCA Reagent B (50∶1, Reagent A: B). Pipette 25 µL of each standard or urine sample (1∶100 dilution) replicate into a microplate well. Add 200 µL of the WR to each well and mix plate thoroughly on a plate shaker for 30 seconds. Cover plate and incubate at 37°C for 30 minutes. Measure the absorbance at 570 nm on a plate reader.

### Cell Sorting

Murine renal tissues were surgical resected and dispersed in RPMI 1640 containing 5% FBS and 0.1% collagenase (Sigma-Aldrich) at 37°C for 30 min, followed by progressive sieving to obtain single-cell suspensions. To analyze gene expression in the renal macrophages, CD11b^+^/F4/80^high^ renal macrophages were sorted from nephritic single-cell suspensions using a FACSAria (BD Biosciences) with FITC-labeled anti-F4/80 and PE-labeled anti-CD11b (BD Biosciences).

### Measurement of GRN Levels

To assess protein levels of GRN in murine serum, ELISA assays were performed with commercial mouse GRN ELISA kit (R&D Systems) according to the manufacturer’s instructions.

### Pathological Analysis

For histology analysis, murine renal tissues were surgical resected and fixed in 4% paraformaldehyde (Sigma-Aldrich), processed, and embedded in paraffin. H&E staining of renal tissue sections were performed according to the manufacturer’s instructions and assessed by two pathologists blinded to treatment group as previously described [22], [25]. The kidney score of glomerulonephritis was determined by using the ISN/RPS2003 classification. Fluorescent staining of cryosections was used for autoantibody deposition analysis in the glomeruli. Sections were fixed in acetone for 10 min and incubated with FITC conjugated goat anti-mouse IgG (H+L chain specific) Ab (Sigma-Aldrich) for 30 min. Pictures were acquired with Nikon SCLIPSS TE2000-S microscope (Nikon) equipped with ACT-1 software (Nikon). Original magnification was ×200.

### Real-time PCR Analysis

Total RNA was isolated from cultured cells or mouse renal macrophages with TRIzol reagent (Invitrogen) and was reversely transcribed using a cDNA synthesis kit (Takara) according to the manufacturer’s instructions. Subsequently, cDNA was subjected to quantitative real-time PCR using a Lightcycler480 and SYBR Green system (Roche Diagnostic Systems) following the manufacturer’s protocol. The primer sequences used in this study were as follows: (1) GRN: 5′-GCTACAGACTTAAGGAACTC-3′ (forward) and 5′-GAAATGGCAG- TTTGATACGG-3′ (reverse); (2) GAPDH: 5′-CTCTGGAAAGCTGTGGCGTGATG-3′ (forward) and 5′-ATGCCAGTGAGCTTCCCGTTCAG-3′ (reverse), GAPDH was used as a housekeeping gene for normalization; (3) TNF-α: 5′-AAGCCTGTAGCCCACGTCGTA-3′ (forward) and 5′-GGCACCACTAGTTGGTTGTCTTTG-3′ (reverse); (4) IL-1β: 5′-TCACAGCAGCACATCAACAA-3′ (forward) and 5′-TGTCCTCATCCTGGAAGGT-3′ (reverse); (5) IL-6∶5′-ACAACCACGGCCTTCCCTACTT-3′ (forward) and 5′-CACG- ATTTCCCAGAGAACATGTG-3′ (reverse); (6) IL-10∶5′-CCAAGCCTTATCGGA- AATGA-3′ (forward) and 5′-TTCACAGGGGAGAAATCG-3′ (reverse); (7) IL-12∶5′-GGAAGCACGGCAGCAGAATA-3′ (forward) and 5′-AACTTGAGGGAGAA- GTAGGAATGG-3′ (reverse); (8) MCP-1∶5′-CCCACTCACCTGCTGCTACT-3′ (forward) and 5′-TCTGGACCCATTCCTTCTTG-3′ (reverse); (9) Nos2∶5′-CCAAGCCC- TCACCTACTTCC-3′ (forward) and 5′-CTCTGAGGGCTGACACAAGG-3′ (reverse); (10) Arg1∶5′-CTCCAAGCCAAAGTCCTTAGAG-3′ (forward) and 5′-AGGAGCTGTCA- TTAGGGACATC-3′ (reverse).

### ELISA Assay

To assess protein levels of TNF-α, IL-1β, IL-6, IL-10, IL-12 and MCP-1 in the cell culture, ELISA assays were performed with relative ELISA Kits (eBioscience) according to the manufacturer’s instructions.

### Western Blot Analysis

The supernatants from macrophages were separated by 10% SDS-PAGE and immunoblotted with an anti-granulin under non-reducing conditions [13]. Extraction protein from cells was measured by the BCA protein assay reagent kit (Thermo Scientific). 50 µg of protein was resolved by 10% SDS-PAGE and immunoblotted with the indicated antibodies with appropriate HRP-conjugated antibodies as secondary antibodies (Cell Signaling Technology) as described previously [29].

### Statistical Analysis

All data are expressed as means ± SD of three independent experiments or from a representative experiment of three independent experiments. Statistical analysis of the data was performed by using the GraphPad Prism (Version 5.0) statistical program. Unpaired Student’s t-test and Pearson correlation analysis were used for statistical analysis. A value of *P*<0.05 was considered statistically significant.

## Results

### Serum GRN Levels were Positively Correlated with the Severity of Lupus Nephritis

**Figure 1:**
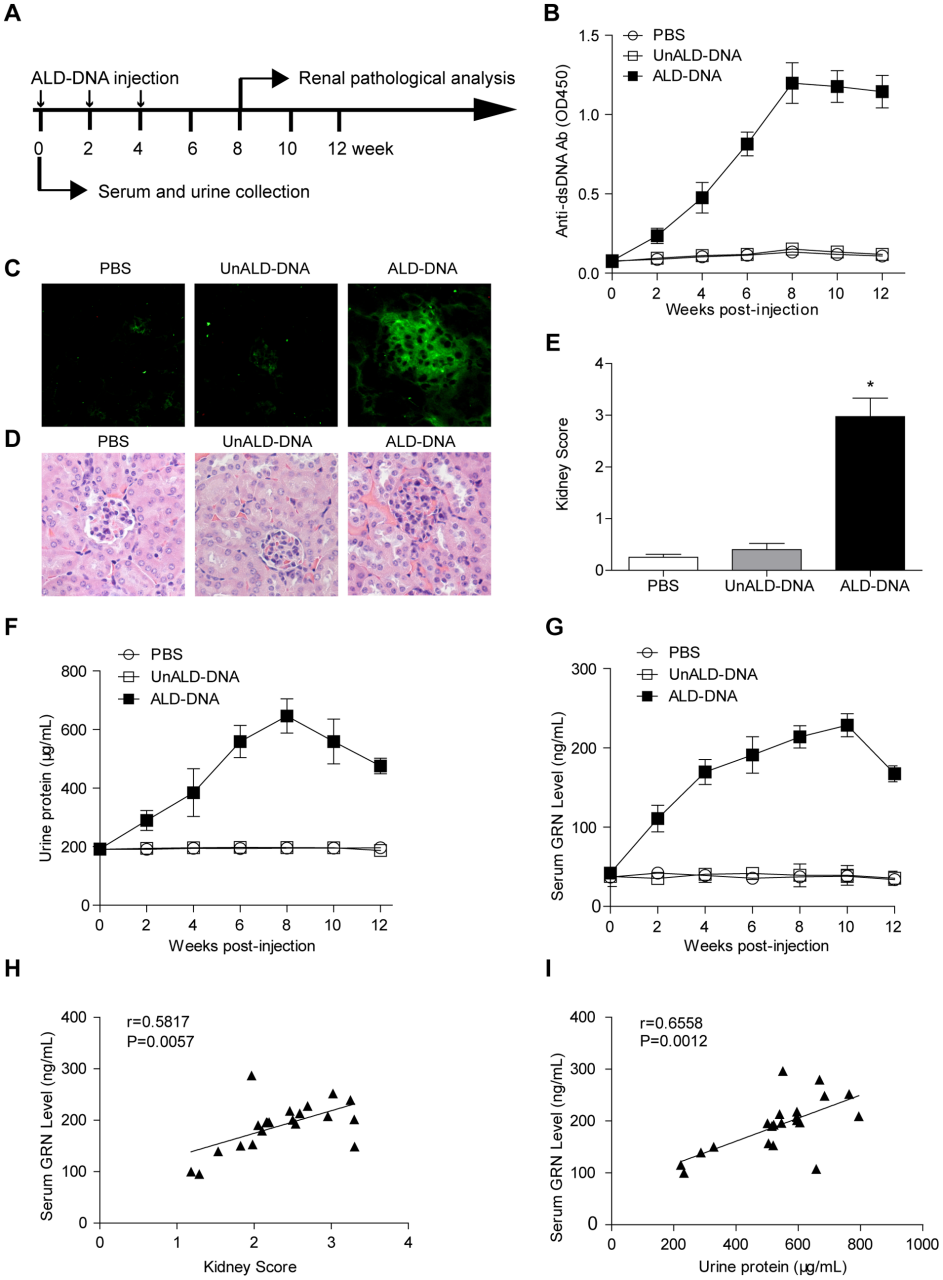
Serum granulin (GRN) levels were up-regulated in lupus model and were associated with the severity of lupus nephritis. (A) Schematic diagram of DNA injection. 6- to 8-week old female BALB/c mice were injected subcutaneously with ALD-DNA (50 µg/mice) plus CFA at week 0, followed by two booster injections of ALD-DNA (50 µg/mice) emulsified with IFA at week 2 and week 4 after initial injection. (B) Serum anti-dsDNA IgG levels were measured by ELISA every 2 weeks after initial injection. Data are means ± SD from 8 mice in each group. (C) 8 weeks after initial injection, glomerular immune deposition were detected by direct immunofluorescence for IgG in frozen kidney section from ALD-DNA-injected SLE mice or control mice. Representative images (magnification×200) of 8 mice are shown for each group. (D) Nephritic pathology was evaluated by H&E staining of renal tissues. Images (magnification×200) are representative of 8 mice in each group. (E) The kidney score was assessed using paraffin sections stained with H&E in (D). n = 8. (F) Urine protein levels of the mice were assessed by BCA Protein Assay Kit every 2 weeks. Data are means ± SD from 8 mice in each group. (G) Serum GRN levels were measured by ELISA every 2 weeks after initial injection. Data are means ± SD from 8 mice in each group. (H) The correlation between serum GRN level and kidney score in lupus model. Correlation analysis was performed by Pearson correlation analysis. Each symbol indicates an individual mouse (n = 21). (I) The correlation between serum GRN level and urine protein level in lupus model. Pearson correlation analysis was used to carry out the correlation study. Each symbol indicates an individual mouse (n = 21). *, *P*<0.05.

Recent report showed that serum GRN levels were elevated in SLE patients in parallel with disease activities, while its potential role in LN, one of the most sever manifestations of SLE, has remained unclear [19]. To study whether GRN was elevated in lupus model, we generated lupus mice according to our previously reported procedure [20]–[22], [24]–[26]. Briefly, female BALB/c mice were injected with ALD-DNA (Fig. 1A), and high titers of anti-dsDNA Abs (Fig. 1B), immune complex glomerular deposition (Fig. 1C), glomerulonephritis (Fig. 1D, 1E) and proteinuria (Fig. 1F) could be detected in this lupus model. Serum GRN levels of lupus model were assayed by ELISA every 2 weeks and we found significantly increased serum GRN levels in lupus mice as compared with those in control mice 4 weeks after the initial ALD-DNA injection (Fig. 1G). Pearson correlation analysis showed that the serum GRN levels were positively correlated with kidney scores (r = 0.5817, *P* = 0.0057) (Fig. 1H), indicating that GRN was significantly correlated with the severity of LN. To further examine the correlation of serum GRN levels with the severity of LN, we then analyzed the correlation of serum GRN levels with urine protein levels. Notably, serum GRN levels were also positively associated with urine protein levels (r = 0.6558, *P* = 0.0012) (Fig. 1I). Taken together, these data indicated that serum GRN levels were up-regulated and were associated with the pathogenesis of LN in lupus mice.

### GRN Could Exacerbate Lupus Nephritis

**Figure 2:**
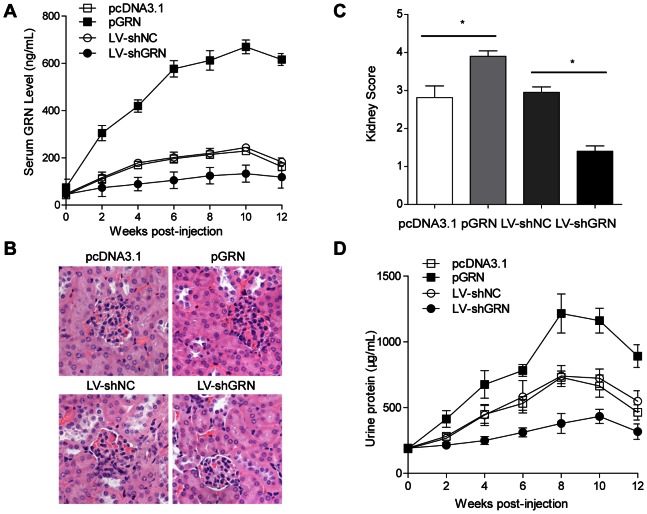
GRN exacerbated lupus nephritis in lupus model. BALB/c mice were administrated intramuscularly with 100 µg/mouse pGRN or pcDNA3.1 to overexpress GRN, intravenously injected with LV-shGRN or LV-shNC (2×10^8^ molecules/mouse) to down-regulate GRN expression. And 72h later, mice were then injected subcutaneously with ALD-DNA (50 µg/mouse) for total 3 times in 4 weeks. (A) The dynamics of serum GRN levels were measured by ELISA every 2 weeks after initial injection. Data are means ± SD from 8 mice in each group. (B) Nephritic pathology was evaluated by H&E staining of renal tissues. Images (magnification×200) are representative of at least 8 mice in each group. (C) The kidney score was assessed using paraffin sections stained with H&E in (B). n = 8. (D) Urine protein levels of the mice were assessed by BCA Protein Assay Kit every 2 weeks. Data are means ± SD from 8 mice in each group. *, *P*<0.05.

Our results described above provided the basis for the hypothesis that GRN administration *in vivo* might modulate the pathogenic process of LN. So we first up-regulated the expression of GRN by injecting BALB/c mice intramuscularly with pGRN as previously described to evaluate whether GRN was critical for the pathogenic process of LN [24]. The serum GRN levels were significantly elevated in pGRN-treated lupus mice compared with those in pcDNA3.1-treated lupus mice (Fig. 2A). To investigate the effect of increased GRN on ALD-DNA-induced LN, we then analyzed renal pathology, kidney score and proteinuria in pGRN-treated or pcDNA3.1-treated lupus mice. Exacerbation of renal pathology was found in the pGRN-treated lupus mice as compared with those controls (Fig. 2B), and the kidney score of pGRN-treated lupus mice was much higher than that of control mice (Fig. 2C), indicating severer LN resulted from GRN overexpression. Furthermore, distinguished increase of urine protein was observed in the pGRN-treated lupus mice as compared with those controls (Fig. 2D). Except for the increased levels of urine protein, we also noticed that overexpression of GRN could accelerate the initiation of proteinuria (Fig. 2D), a strong evidence for the aggravated LN by GRN overexpression. We further detected serum creatinine level, a commonly accepted index of kidney function [30], in pGRN-treated or pcDNA3.1-treated lupus mice. Serum creatinine levels were up-regulated in pGRN-treated lupus mice (Fig. S1), indicating more sever LN by GRN overexpression.

To further confirm the modulation effect of GRN on the initiation and progression of LN, we then investigated whether down-regulation of GRN could ameliorate LN in lupus model. We down-regulated the levels of GRN *in vivo* by intravenous injection of lentivirus encoding shRNA directed against GRN (LV-shGRN) or lentivirus encoding nontargeting shRNA (LV-shNC) as control. Decreased serum GRN levels were found in lupus mice injected with LV-shGRN versus those injected with LV-shNC (Fig. 2A). We then measured the renal tissue H&E staining, kidney score and urine protein to examine whether down-regulation of GRN could ameliorate LN. Notably, we observed that renal pathology was ameliorated in LV-shGRN-injected lupus model versus controls (Fig. 2B). We further analyzed the kidney score and observed that LV-shGRN-injected lupus mice got lower kidney score compared with control mice (Fig. 2C), suggesting that down-regulation of GRN could ameliorate LN in lupus model. Moreover, urine protein showed delayed onset as well as remarkable reduction in LV-shGRN-injected lupus mice compared with those of control mice (Fig. 2D). And LV-shGRN-injected lupus mice had a lower serum creatinine level compared with control mice (Fig. S1). These findings presented above collectively showed that GRN could aggravate LN in lupus mice.

### GRN Exacerbated Lupus Nephritis by Enhancing Macrophage M2b Polarization

**Figure 3:**
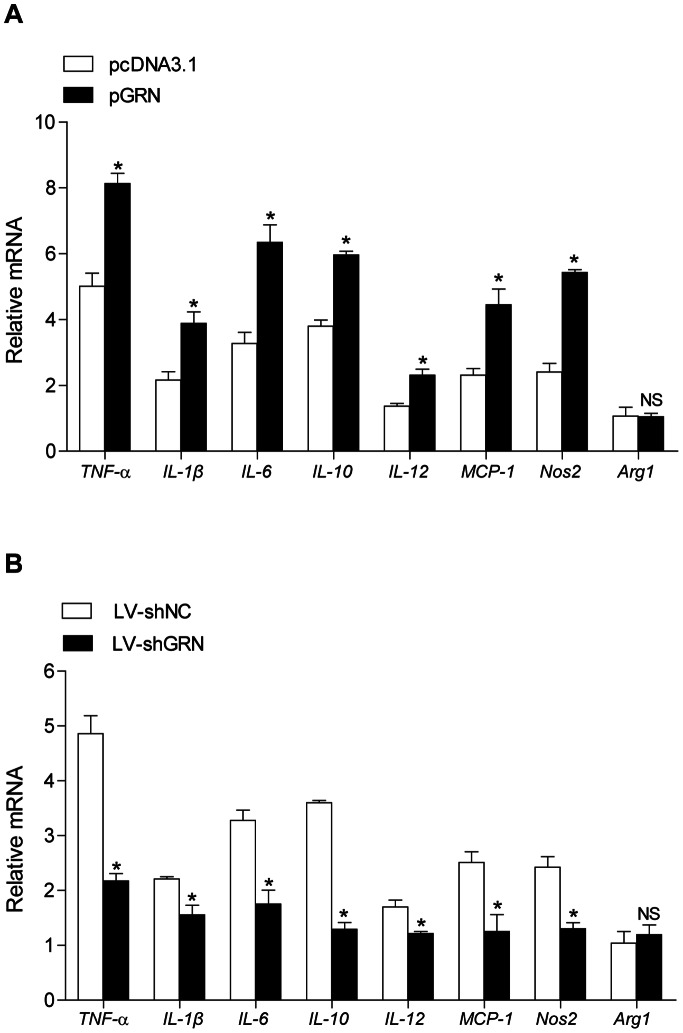
GRN aggravated lupus nephritis in lupus model by enhancing ALD-DNA-induced M2b polarization. CD11b^+^/F4/80^high^ renal macrophages were sorted from nephritic single-cell suspensions from pGRN-treated, pcDNA3.1-treated, or LV-shGRN-injected, LV-shNC-injected lupus model by flow cytometry. (A) mRNA levels of *TNF-α*, *IL-1β*, *IL-6*, *IL-10*, *IL-12*, *MCP-1*, *Nos2 (iNOS)* and *Agr1* in the renal macrophages screened from pGRN or pcDNA3.1-treated lupus mice were evaluated by real-time PCR. Data are means ± SD from 8 mice in each group. (B) Real time analysis for the mRNA levels of *TNF-α*, *IL-1β*, *IL-6*, *IL-10*, *IL-12*, *MCP-1*, *Nos2 (iNOS)* and *Agr1* in renal macrophages from LV-shGRN-injected lupus mice and control mice. Data are means ± SD from 8 mice in each group. *, *P*<0.05.

Macrophage activation has been found in autoimmune and inflammatory diseases and in LN in particular [31]. Of note, M2b macrophages served as key mediators of the initiation and progression of LN [32], [33]. Consistently, our previous work showed that ALD-DNA-induced M2b-differentiated renal macrophages mediated the initiation and progression of LN [22], [25]. Besides, GRN could enhance the innate immune response in macrophages [11], [13]. To test whether GRN could aggravate LN by enhancing ALD-DNA-induced M2b-polarized macrophages, we first isolated CD11b^+^/F4/80^high^ renal macrophages from pGRN or pcDNA- 3.1-treated lupus mice, LV-shGRN or LV-shNC-injected lupus mice. Real time analysis was performed to detect the gene expression levels of *TNF-α*, *IL-1β*, *IL-6*, *IL-10*, *IL-12*, *MCP-1* and *Nos2 (iNOS)*, representing M2b-polarized phenotype, as well as *Arg1*, a marker for M2a and M2c macrophages [34]. Although renal macrophages from pcDNA3.1-treated lupus mice exhibited enhanced mRNA levels of *TNF-α*, *IL-1β*, *IL-6*, *IL-10*, *MCP-1* and *Nos2*, macrophages from pGRN-treated mice showed much higher expression levels of *TNF-α*, *IL-1β*, *IL-6*, *IL-10*, *MCP-1* and *Nos2*, slightly higher *IL-12* and no significant changes in the expression of *Arg1* (Fig. 3A), suggesting that up-regulated expression of GRN promoted renal macrophages toward M2b differentiation induced by ALD-DNA. In contrast, administration with LV-shGRN in lupus model considerably inhibited M2b polarization, as renal macrophages from LV-shGRN-injected lupus mice performed lower mRNA levels of *TNF-α*, *IL-1β*, *IL-6*, *IL-10*, *IL-12*, *MCP-1* and *Nos2* than those of controls (Fig. 3B), confirming the reinforced effect of GRN on ALD-DNA-induced M2b polarization. Consequently, these results demonstrated that GRN exacerbated LN in lupus model by promoting ALD-DNA-induced M2b polarization.

### GRN Promoted ALD-DNA-induced M2b Polarization *in vitro*

**Figure 4:**
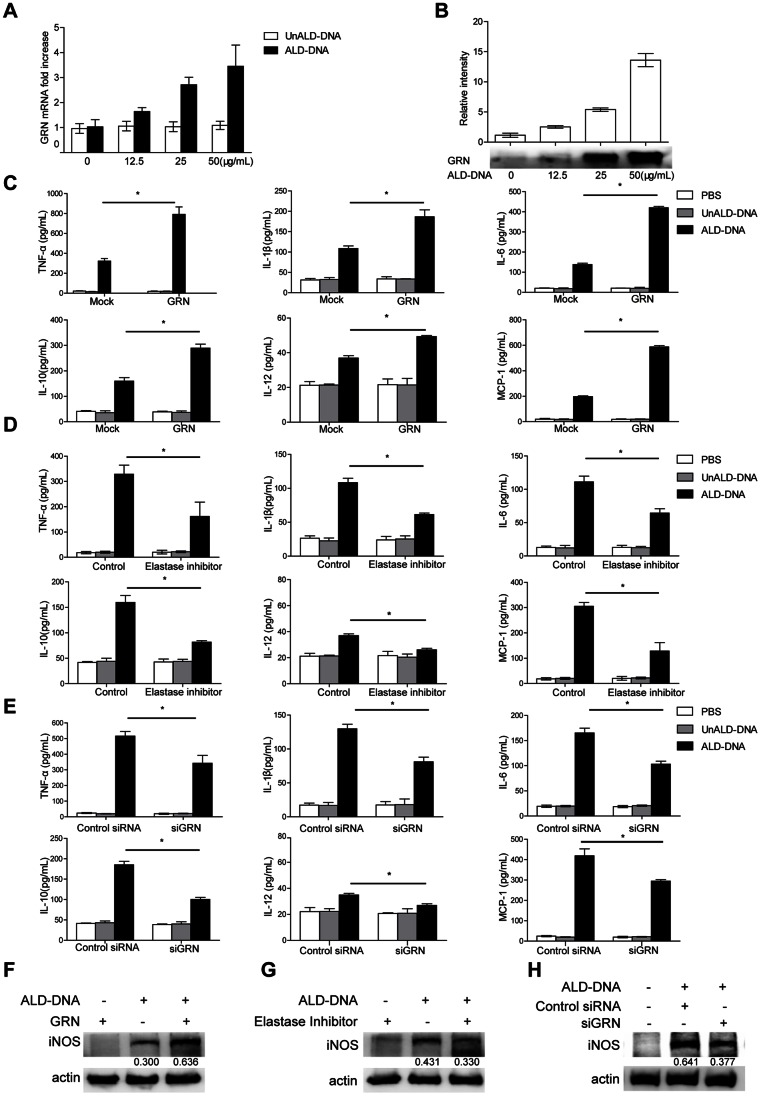
GRN played a pivotal role in the ALD-DNA-induced M2b polarization *in vitro*. (A–B) Primary macrophages were stimulated with increasing amounts of ALD-DNA for 24 h. mRNA levels of GRN in macrophages were analyzed by real time PCR analysis (A), and protein levels of GRN in the supernatants of macrophages were analyzed by western blot (B). Above, quantitative results of western blots, the band intensity was measured by Image J; Below, representative western blots. Similar results were obtained in three independent experiments. Data are representative of results obtained in three independent experiments. Primary peritoneal macrophages were stimulated by ALD-DNA (50 µg/mL) with GRN (5 µg/mL) for 24 h (C and F), or were pretreated with elastase inhibitor (100 µM) or DMSO (0.1%) for 12 h (D and G), and then were exposed to ALD-DNA, UnALD-DNA, or PBS for another 24 h. Macrophages were transfected with control siRNA (200 nM) or GRN siRNA (siGRN, 200 nM). 36 h posttransfection, macrophages were stimulated with PBS, UnALD-DNA or ALD-DNA (50 µg/mL) (E and H). (C–E) ELISA assay was used to analyze the levels of TNF-α, IL-1β, IL-6, IL-10, IL-12, and MCP-1 in the culture supernatants of macrophages. Data are means ± SD of three independent experiments. (F–H) Western blot analysis was used to analyze the protein levels of iNOS in macrophages. Data are representative of three separate experiments. Similar results were obtained in three independent experiments. Band intensity was measured by Image J and the ratios of iNOS to β-actin were calculated. *, *P*<0.05.

We then investigated whether GRN played a crucial role in ALD-DNA-triggered M2b differentiation *in vitro*. Consistent with our previous studies, significantly high supernatant levels of TNF-α, IL-1β, IL-6, IL-10, and MCP-1 but not IL-12, and induced expression of iNOS were found in macrophages when stimulated with ALD-DNA (Fig. S2A, S2B), indicating that macrophages treated with ALD-DNA displayed the M2b phenotype *in vitro*. Further analysis of M2b-polarized macrophages showed increased GRN mRNA levels after ALD-DNA treatment in dose-dependent manner (Fig. 4A). Consistently, protein levels of GRN in supernatants were also elevated in dose-dependent manner (Fig. 4B). These data suggested that ALD-DNA could effectively enhance the expression of GRN both in mRNA levels and protein levels in M2b macrophage *in vitro*.

To explore the possible contribution of GRN to ALD-DNA-induced M2b differentiation *in vitro*, we added purified GRN to the culture medium in which macrophages were maintained, and then stimulated macrophages with ALD-DNA. ELISA analysis for the cytokine markers evoked by ALD-DNA showed remarkably increased production of TNF-α, IL-1β, IL-6, IL-10, IL-12, and MCP-1 compared with those in controls (Fig. 4C). Moreover, we also found that addition of purified GRN considerably up-regulated the expression of iNOS in macrophages (Fig. 4F). Meanwhile, GRN could also promote the expression of TNF-α, IL-6 and IL-10 induced by ALD-DNA in dose-dependent manner (Fig. S3). Thus, these results suggested the significant role of GRN in potentiating ALD-DNA-induced M2b polarization.

Elastase inhibitor was used to blunt the generation of GRN [12], [13], [35]. To test whether GRN was required for ALD-DNA-elicited M2b polarization, we exposed macrophages to an elastase-selective inhibitor for 12 h and then stimulated macrophages with ALD-DNA. Inclusion of elastase inhibitor interfered with ALD-DNA-triggered M2b polarization, verified by the decreased supernatant levels of TNF-α, IL-1β, IL-6, IL-10, IL-12, and MCP-1 (Fig. 4D), as well as the reduced protein levels of iNOS in macrophages (Fig. 4G). To further validate the effect of GRN in facilitating ALD-DNA-induced macrophage M2b polarization, we down-regulated GRN expression in macrophages with specific GRN siRNA (siGRN), the knockdown efficiency was shown in Figure S4A and S4B. 36 h posttransfection, we then stimulated macrophages with ALD-DNA and noticed that down-regulation of GRN effectively reduced the supernatant levels of TNF-α, IL-1β, IL-6, IL-10, IL-12, and MCP-1 compared with those in controls (Fig. 4E), accompanied with decreased iNOS by western blot analysis (Fig. 4H). Taken together, we concluded that GRN was pivotal for ALD-DNA-induced M2b polarization.

### MAPK Signaling Pathways were Required for GRN to Facilitate ALD-DNA-induced M2b Polarization

**Figure 5:**
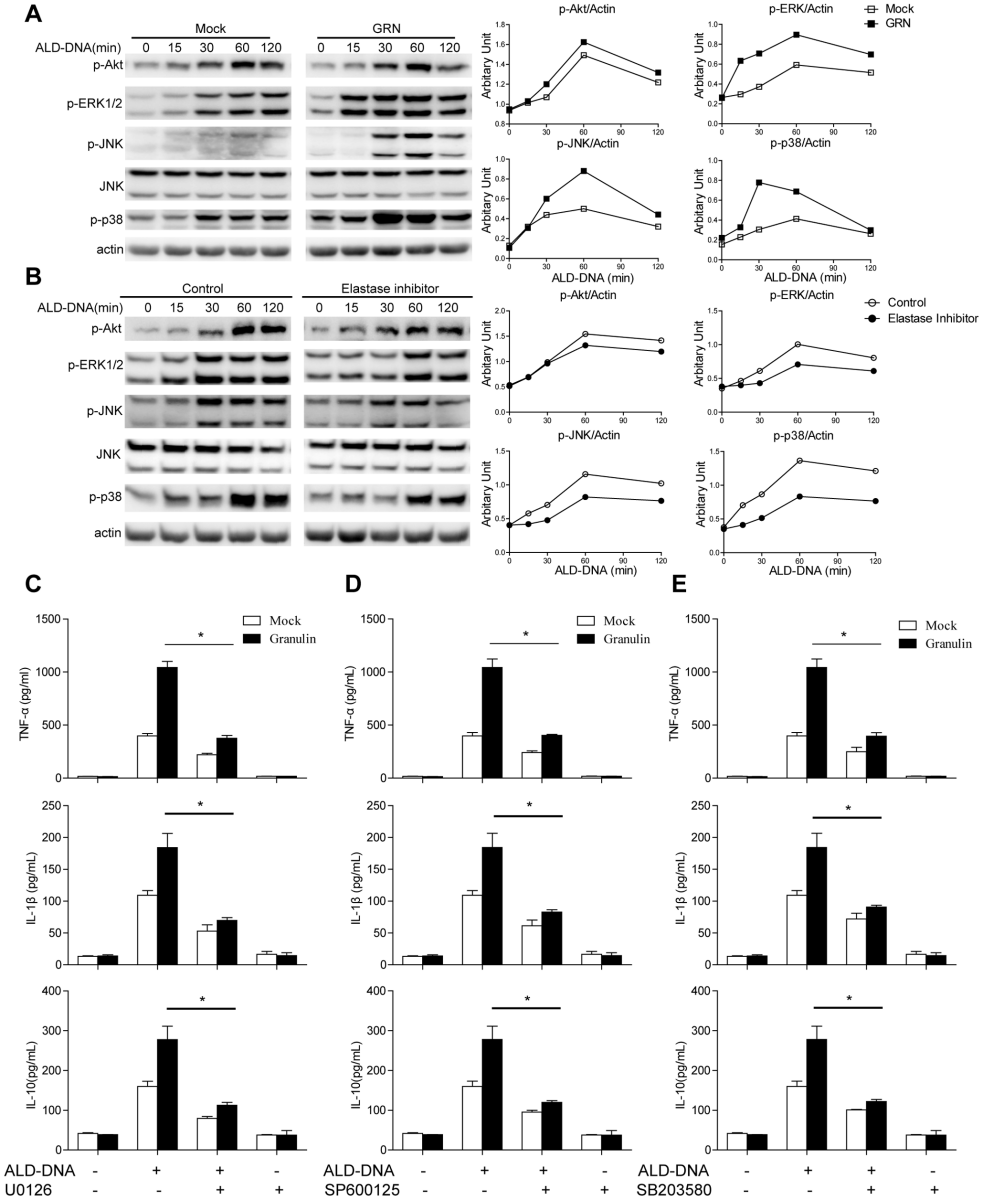
GRN modulated ALD-DNA–induced M2b polarization via MAPK signaling pathway. (A) Primary macrophages were stimulated with 50 µg/mL ALD-DNA together with 5 µg/mL purified GRN for the indicated time. Phospho-Akt, ERK, JNK, and p38 were detected by immunoblotting. Data are representative of three separate experiments. Similar results were obtained in three independent experiments. (B) Primary macrophages were pretreated with elastase inhibitor (100 µM) for 12 h, and then were stimulated with 50 µg/mL ALD-DNA for the indicated time. Phospho-Akt, ERK, JNK, and p38 were detected by Western blot. Data are representative of three separate experiments. Similar results were obtained in three independent experiments. Left, representative western blots; Right, quantitative results, the band intensity was measured by Image J and the ratios of phospho-Akt, phospho-ERK, phospho-JNK and phospho-p38 to β-actin were calculated. Primary peritoneal macrophages were pretreated with U0126 (10 µM) (C), SP600125 (10 µM) (D), SB203580 (10 µM) (E) for 30 min, and then were stimulated with 50 µg/mL ALD-DNA together with 5 µg/mL purified GRN for 24 h. Cytokine expression levels of TNF-α, IL-1β and IL-10 in the supernatants of macrophages were determined by ELISA assay. Data are means ± SD of three independent experiments. *, *P*<0.05.

Previous reports demonstrated that GRN exerted its multiple effects through activating both MAPK and PI3K/Akt pathways [16], [36]–[38]. We then investigated these major intracellular signaling pathways (Akt, ERK 1/2, JNK, p38) as possible mediators of GRN in regulation of ALD-DNA-induced macrophages M2b polarization. We found that whereas ALD-DNA stimulation alone effectively induced the phosphorylation of Akt, ERK1/2, JNK, and p38, addition of purified GRN strikingly enhanced ALD-DNA-induced ERK1/2, JNK, and p38 phosphorylation compared with mock (Fig. 5A). In contrast, there was no appreciable increase in phosphorylation of Akt between macrophages treated with GRN and mock (Fig. 5A). On the contrary, exposure to elastase inhibitor abrogated the ALD-DNA-induced activation of ERK1/2, JNK, and p38 signal, while leaving the phosphorylation of Akt unaffected (Fig. 5B). Furthermore, macrophages transfected with GRN specific siRNA showed the expected attenuation of phosphorylated ERK1/2, JNK, and p38 and unimpaired phosphorylation of Akt (Fig. S4C). Band density quantification of these results is shown in Figure 5A, 5B, and S4C.

To further explore whether MAPK signals were required for the effect of GRN on ALD-DNA-induced macrophage M2b polarization, we pretreated macrophages with specific MEK1/2 inhibitor U0126, JNK inhibitor SP600125, or p38 MAPK inhibitor SB203580 for 30 min before ALD-DNA stimulation and then explored the effect of GRN on ALD-DNA-triggered M2b polarization. The results showed that blockade of ERK1/2, JNK, or p38 activation dramatically blunted the increase in GRN supplements-mediated TNF-α, IL-1β and IL-10 production (Fig. 5C, 5D, 5E), indicating that the activation of MAPK signaling pathways were required for the effect of GRN on ALD-DNA-induced M2b polarization.

## Discussion

In earlier studies, GRN was reported as a multifaceted growth factor that played an important role in the maintenance and regulation of the homeostatic dynamics of normal tissue development, proliferation, regeneration, and the host-defense response [39]–[42]. Recently, it was reported that GRN played crucial roles in inflammation and immunoregulation. GRN-deficient mice failed to clear *Listeria monocytogenes* infection and allowed bacteria to proliferate [11], and also showed a substantial reduction in serum amounts of TNF-α and IL-6 upon administration of CpG-ODNs [13]. Furthermore, the elevation of GRN levels was observed in local inflammatory tissues of patients with autoimmune diseases, such as brains in patients with active multiple sclerosis [17], and synovium of rheumatoid arthritis patients [43]. Until recently, one study reported that GRN was extensively correlated with the disease activity of SLE [19]. All these findings indicated that GRN might be involved in the pathogenesis of LN, one of the most severe manifestations of SLE. In our present study, we first demonstrated that GRN was intensively involved in the pathogenesis of LN. We found that serum GRN level was positively correlated with kidney score as well as urine protein level. Furthermore, we also demonstrated that overexpression of GRN exacerbated LN, whereas down-regulation of GRN could ameliorate LN, verifying that GRN could exacerbate LN.

Much of the pathophysiology and therapy of SLE has focused on autoimmune B cells and T cells of the adaptive immune system. Recently, studies have also focused on the role of the innate immune system in SLE pathogenesis. In a recent work, a mouse model of an autoimmune disease similar to lupus was reported to be adaptive immune system independent but instead triggered by the innate immune response [31]. As essential components of the innate immune system, macrophages have been found to be activated in inflammatory autoimmune diseases, in particular in LN [31]. Plasticity and flexibility are key features of macrophages and of their activation states, since spatial and temporal differences of microenvironment change the phenotype of macrophages as necessary [44]. Functional macrophage polarization represents different extremes of a continuum ranging from M1, M2a, and M2b to M2c [34]. Classically activated or M1 macrophages are induced by IFN-γ in concert with microbial stimuli. M1 macrophages have an IL-12^high^ IL-10^low^ phenotype, produce high amounts of inflammatory cytokines, and participate in Th1 responses [44], [45]. Alternatively activated or M2a macrophages are induced by Th2 cytokines IL-4 and IL-13. These population of macrophages displays a CD163^+^ CD206^+^ IL-12^low^ IL-10^high^ phenotype, produces ornithine and polyamines via arginase [46], and participates in Th2 responses with immunoregulatory functions [44]–[46], whereas M2c polarization, induced by IL-10, gains immunosuppression and tissue-remodeling activities [34]. Regulatory or M2b macrophages, induced by exposure to ICs and agonists of Toll-like receptors (TLRs) or IL-1R [44], [45], [47], more closely resemble M1 than M2a macrophages for the production of TNF-α and NO, low/no arginase activity and high expression of CD86 and MHC-II [48], [49]. Distinct from M1 macrophages, M2b macrophages express an IL-12^low^ IL-10^high^ phenotype and support the differentiation of Th2 cells [48]. Recently, M2b macrophage levels have been shown to directly correlate with relapse and remission in murine LN [32]. For example, M2b-polarized macrophages release large amounts of pro-inflammatory cytokines, which promote differentiation of arriving monocytes toward the pro-inflammatory phenotype and amplify intrarenal inflammation and loss of renal parenchymal cells in LN [50]. In our recent work, ALD-DNA-induced macrophage M2b differentiation was found to play pivotal role in the pathogenesis of LN [22], [25]. Herein, we found that GRN enhanced the ALD-DNA-induced M2b functional polarization both *in vivo* and *in vitro.* Overexpression of GRN could remarkably up-regulate the ALD-DNA-induced expression of TNF-α, IL-1β, IL-6, IL-10 and MCP-1 as well as iNOS in macrophages, which revealed that enhanced expression of GRN could promote the ALD-DNA-induced M2b functional polarization. On the other hand, down-regulation of GRN could blunt macrophage M2b polarization. We concluded that GRN exacerbated LN by promoting ALD-DNA-induced macrophage M2b polarization. However, the plasticity of macrophages makes the task of assigning a particular phenotype to a particular population of macrophages with specific biochemical markers difficult [22], [25], [44]. The phenotype of renal macrophages isolated *in vivo* and macrophages stimulated by ALD-DNA *in vitro* in this study might be flexible. Relative levels of infiltration by inflammatory and alternatively activated macrophages might change over time during the process of lupus nephritis, and all these macrophage subtypes might coexist within the renal tissue of lupus model, which needs further investigation.

In fact, the role of GRN in other immune cells has also been reported. GRN was reported to promote CD4^+^ T cells to differentiate into Foxp3^+^ Tregs [18], and GRN was also found to be highly expressed in a subpopulation of neutrophils that could promote antibody diversity in B cells [51]. It was also shown that GRN could bind to HLA-DR 11 αβ complex in the B lymphoblastoid cell line, indicating that GRN was involved in the antigen-presenting process [52]. The tempting effects of GRN on these immune cells need our further investigation.

GRN has been reported to exert its multiple effects through the stimulation of both MAPK and the PI3K/Akt pathways [16], [36]–[38]. And in our previous work, PI3K/Akt and MAPK signaling pathways were proved to be involved in ALD-DNA-induced M2b polarization [22], [25]. And in this study, we demonstrated that MAPK signal activation was crucial for GRN to promote ALD-DNA-induced macrophage M2b polarization. The mechanisms of how GRN regulates ALD-DNA-induced MAPK signal activations are studied under way.

Collectively, our study first demonstrated that GRN aggravated ALD-DNA-induced LN via facilitating macrophage M2b polarization, and LN could be ameliorated by down-regulating the expression of GRN in lupus model, thus bringing an insight into better understanding of the underlying mechanism of LN and providing a potential target for clinical therapy.

## Supporting Information

Figure S1 **Serum creatinine levels in pGRN-treated, pcDNA3.1-treated, or LV-shGRN-injected, LV-shNC-injected lupus model.** Serum creatinine levels in pGRN-treated, pcDNA3.1-treated, or LV-shGRN-injected, LV-shNC-injected lupus model were measured by ELISA. Data are means ± SD from 8 mice in each group. *, *P*<0.05.
(TIF)

Figure S2 **ALD-DNA stimulation could induce macrophage M2b polarization** ***in vitro*** **.** Peritoneal macrophages were stimulated with PBS, UnALD-DNA, ALD-DNA (50 µg/mL) for 24 h. (A) Cytokine expression levels of TNF-α, IL-1β, IL-6, IL-10, IL-12, and MCP-1 in the supernatants of peritoneal macrophages were measured by ELISA assay. Data are means ± SD of three independent experiments. (B) Protein levels of iNOS in peritoneal macrophages were analyzed by Western blot analysis. Data are representative of three separate experiments. Similar results were obtained in three independent experiments. *, *P*<0.05.
(TIF)

Figure S3 **GRN could potentiate ALD-DNA-induced M2b polarization in dose-dependent manner** ***in vitro*** **.** Peritoneal macrophages were stimulated with 50 µg/mL ALD-DNA with increasing amounts of GRN as indicated. 24 h poststimulation, the supernatant levels of TNF-α, IL-6 and IL-10 was measured by ELISA analysis. Data are means ± SD of three independent experiments.
(TIF)

Figure S4 **Down-regulation of GRN by siGRN could inhibit ALD-DNA-induced MAPK signal activation.** Macrophages were transfected with control siRNA (200 nM) or GRN siRNA (siGRN, 200 nM). (A) The knockdown efficiency of siGRN in macrophages was analyzed by real time PCR analysis. Data are means ± SD of three independent experiments. (B) The knockdown efficiency of siGRN in macrophages was analyzed by immunoblotting analysis. Above, quantitative results of western blots, the band intensity was measured by Image J; Below, representative western blots. Data are representative of three separate experiments. Similar results were obtained in three independent experiments. (C) 36 h posttransfection, macrophages were stimulated with ALD-DNA (50 µg/mL) for the indicated time. Phospho-Akt, ERK, JNK, and p38 were detected by Western blot analysis. Data are representative of three separate experiments. Similar results were obtained in three independent experiments. Above, representative western blots; Below, quantitative results, the band intensity was measured by Image J and the ratios of phospho-Akt, phospho-ERK, phospho-JNK and phospho-p38 to β-actin were calculated. *, *P*<0.05.
(TIF)
