# Supplementary figures and images for: Granulin Exacerbates Lupus Nephritis via Enhancing Macrophage M2b Polarization
Source: PLoS One. 2013 Jun 5;8(6):e65542. doi: 10.1371/journal.pone.0065542 (PMC3673914; doi:10.1371/journal.pone.0065542)

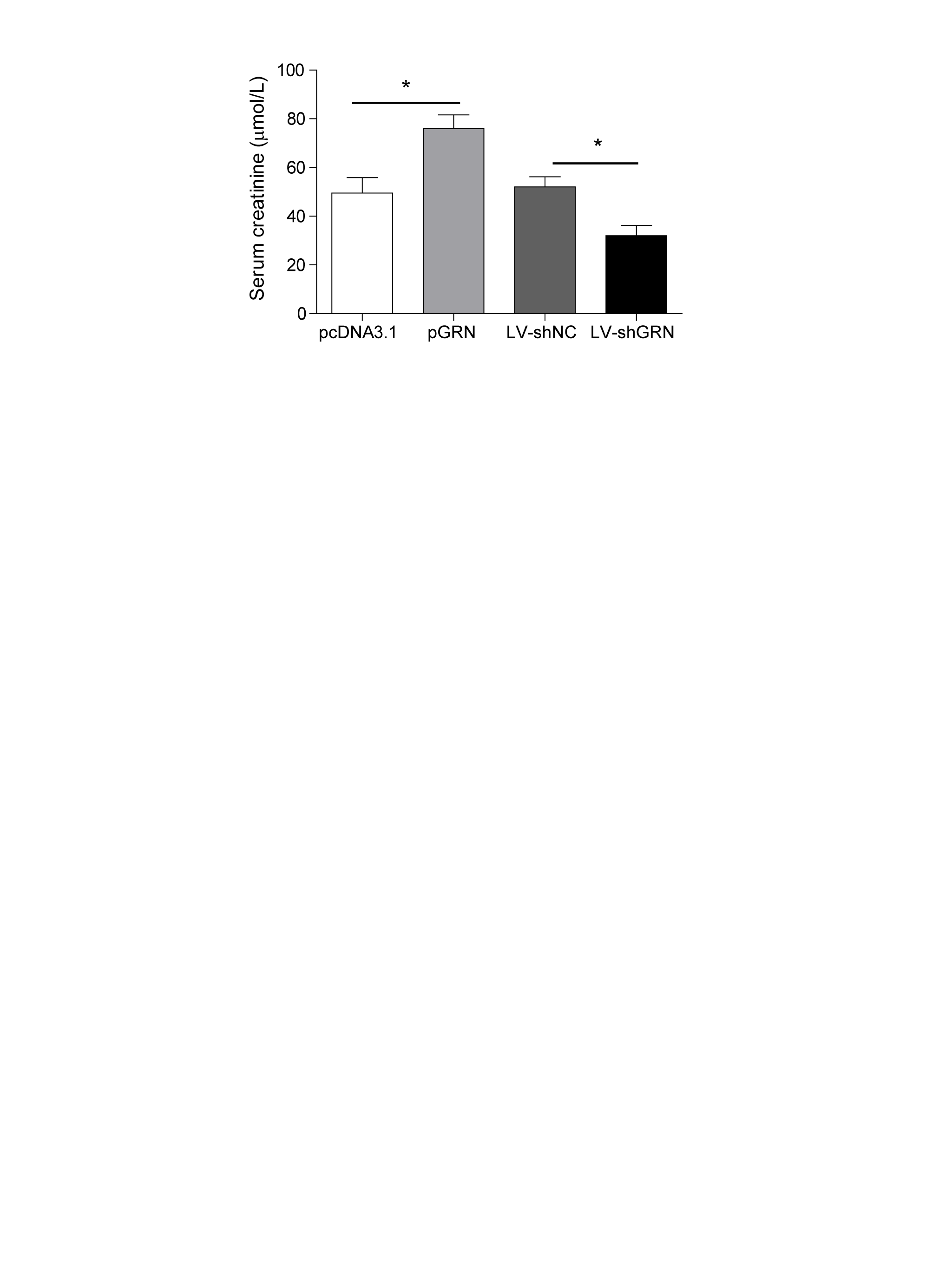

Supplement: Figure S1 — Serum creatinine levels in pGRN-treated, pcDNA3.1-treated, or LV-shGRN-injected, LV-shNC-injected lupus model. Serum creatinine levels in pGRN-treated, pcDNA3.1-treated, or LV-shGRN-injected, LV-shNC-injected lupus model were measured by ELISA. Data are means ± SD from 8 mice in each group. *, P<0.05. (TIF) [file pone.0065542.s001.tif]

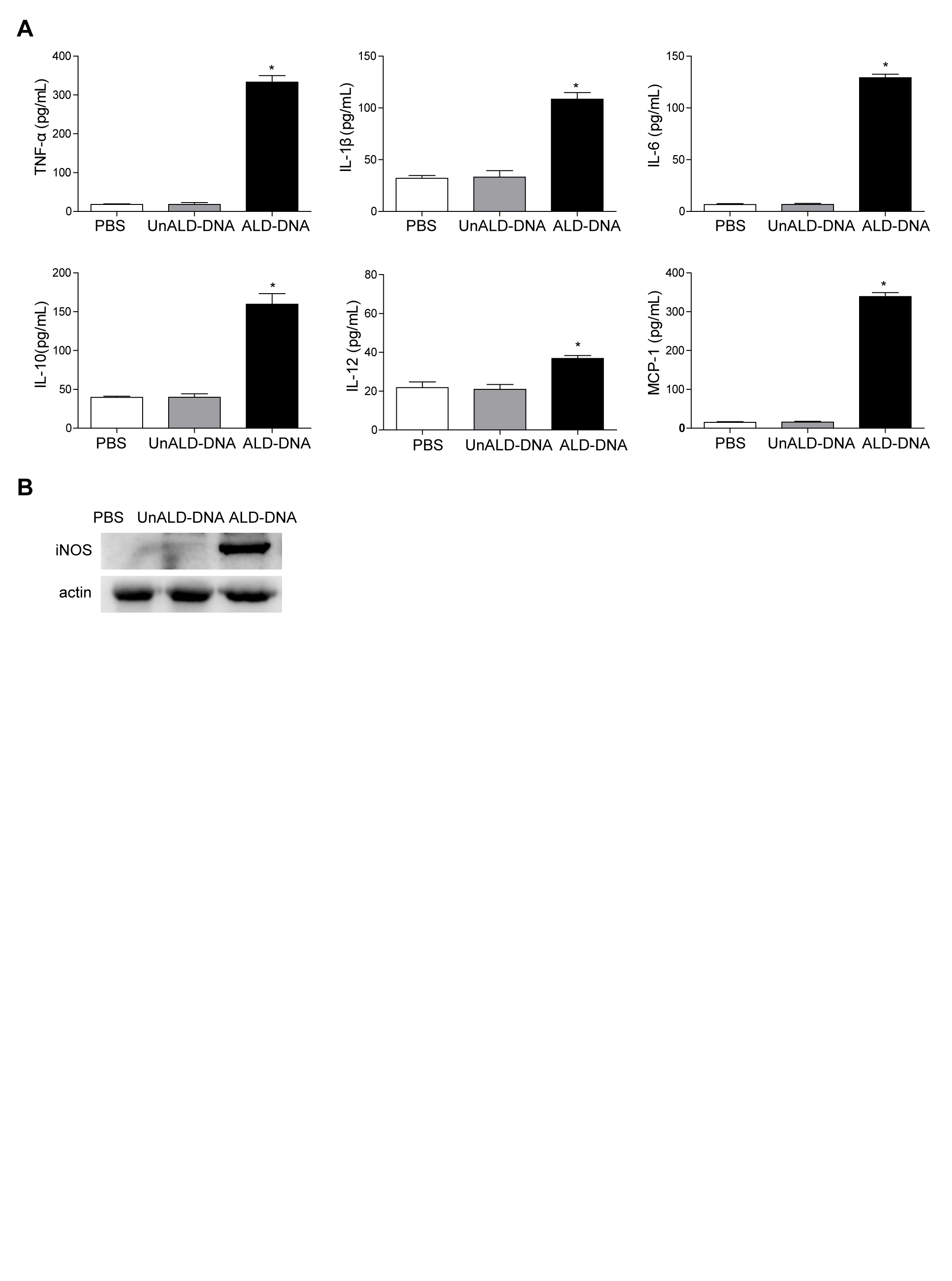

Supplement: Figure S2 — ALD-DNA stimulation could induce macrophage M2b polarization in vitro . Peritoneal macrophages were stimulated with PBS, UnALD-DNA, ALD-DNA (50 µg/mL) for 24 h. (A) Cytokine expression levels of TNF-α, IL-1β, IL-6, IL-10, IL-12, and MCP-1 in the supernatants of peritoneal macrophages were measured by ELISA assay. Data are means ± SD of three independent experiments. (B) Protein levels of iNOS in peritoneal macrophages were analyzed by Western blot analysis. Data are representative of three separate experiments. Similar results were obtained in three independent experiments. *, P<0.05. (TIF) [file pone.0065542.s002.tif]

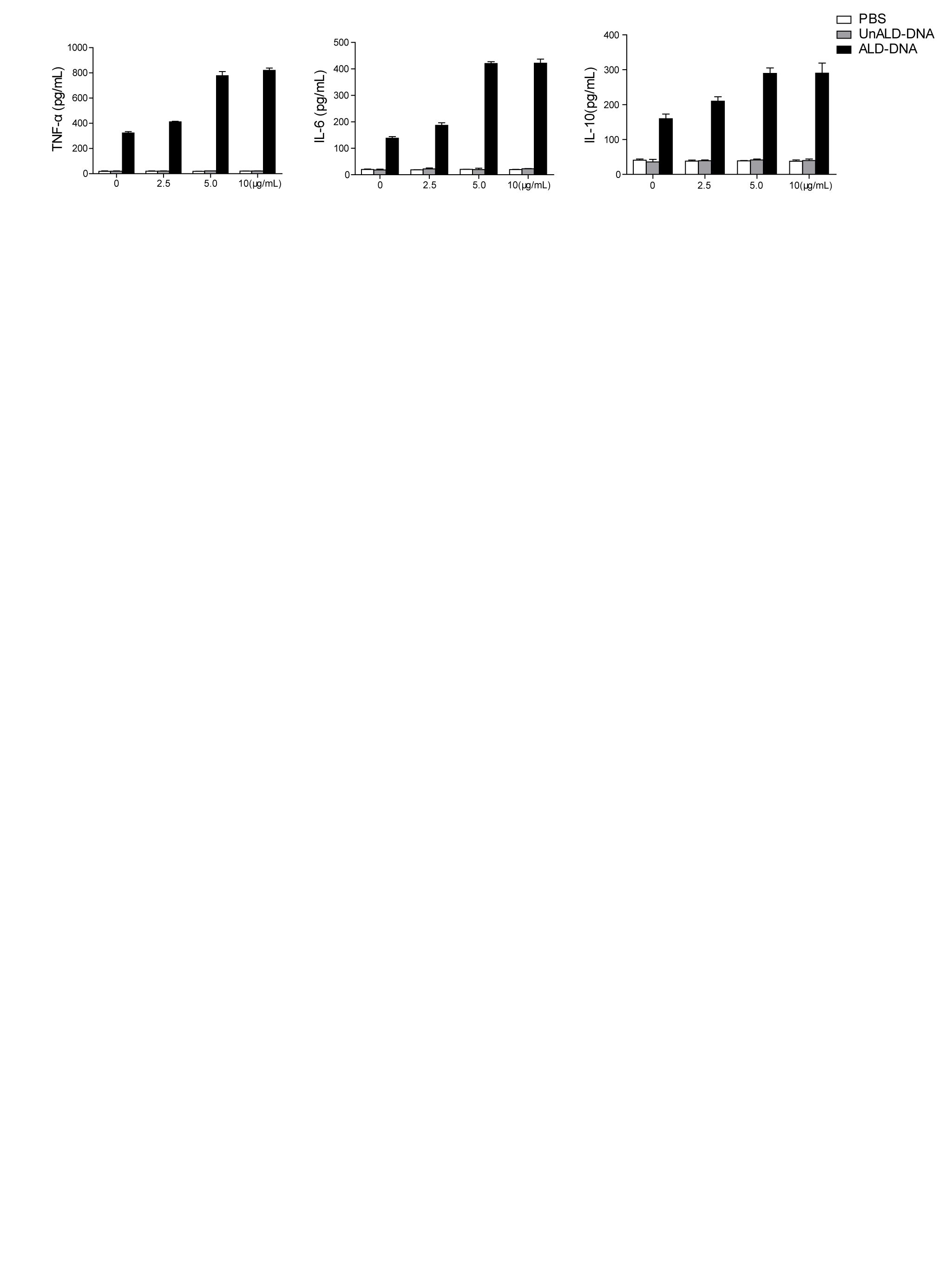

Supplement: Figure S3 — GRN could potentiate ALD-DNA-induced M2b polarization in dose-dependent manner in vitro . Peritoneal macrophages were stimulated with 50 µg/mL ALD-DNA with increasing amounts of GRN as indicated. 24 h poststimulation, the supernatant levels of TNF-α, IL-6 and IL-10 was measured by ELISA analysis. Data are means ± SD of three independent experiments. (TIF) [file pone.0065542.s003.tif]

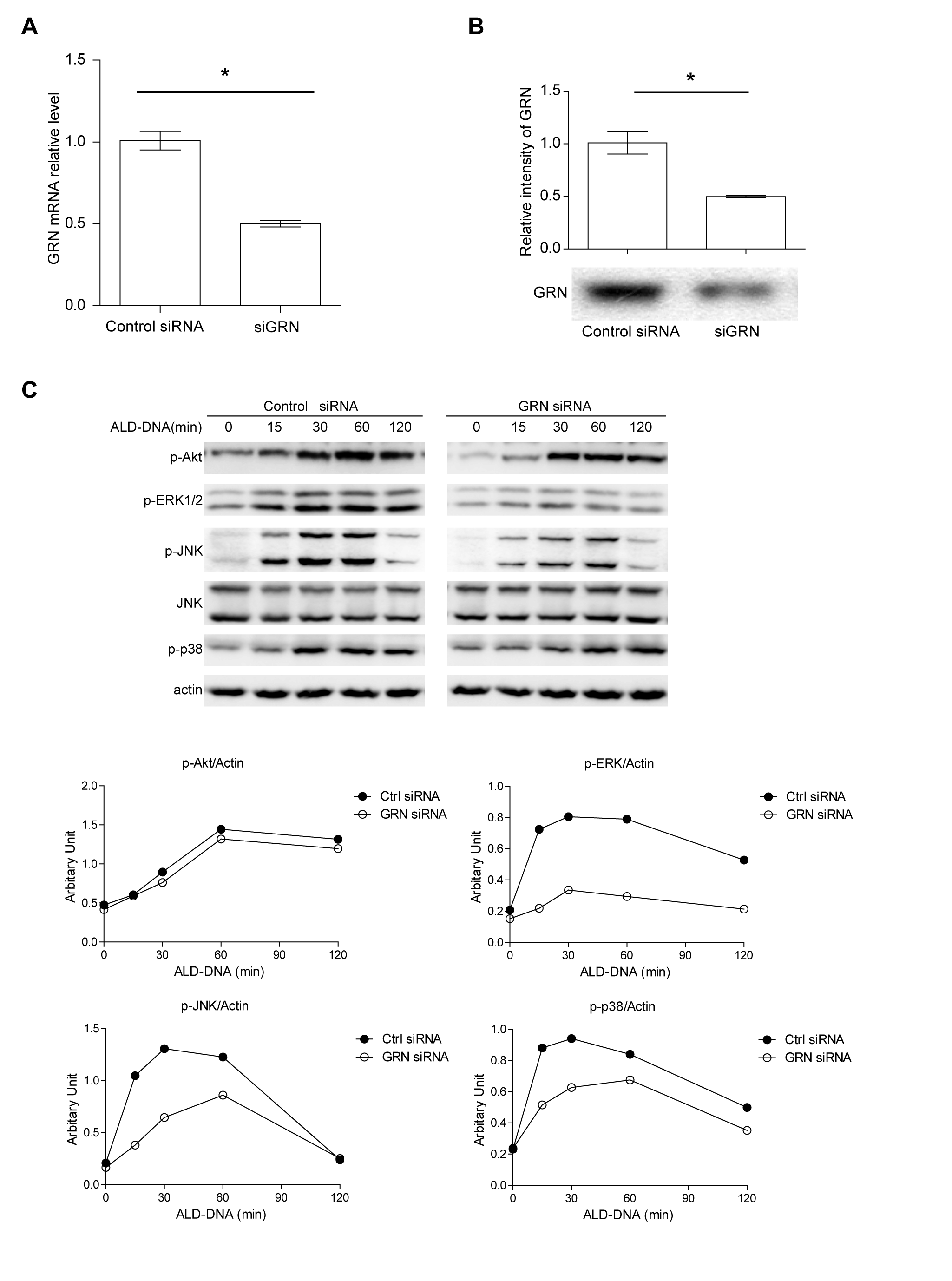

Supplement: Figure S4 — Down-regulation of GRN by siGRN could inhibit ALD-DNA-induced MAPK signal activation. Macrophages were transfected with control siRNA (200 nM) or GRN siRNA (siGRN, 200 nM). (A) The knockdown efficiency of siGRN in macrophages was analyzed by real time PCR analysis. Data are means ± SD of three independent experiments. (B) The knockdown efficiency of siGRN in macrophages was analyzed by immunoblotting analysis. Above, quantitative results of western blots, the band intensity was measured by Image J; Below, representative western blots. Data are representative of three separate experiments. Similar results were obtained in three independent experiments. (C) 36 h posttransfection, macrophages were stimulated with ALD-DNA (50 µg/mL) for the indicated time. Phospho-Akt, ERK, JNK, and p38 were detected by Western blot analysis. Data are representative of three separate experiments. Similar results were obtained in three independent experiments. Above, representative western blots; Below, quantitative results, the band intensity was measured by Image J and the ratios of phospho-Akt, phospho-ERK, phospho-JNK and phospho-p38 to β-actin were calculated. *, P<0.05. (TIF) [file pone.0065542.s004.tif]
